# Pharmaco-Epigenetics and Epigenetic Drugs in Type 2 Diabetes: Can Epigenetics Predict Drug Efficiency?

**DOI:** 10.3390/biomedicines13092278

**Published:** 2025-09-16

**Authors:** Senzosenkosi Surprise Mkhize, Anil Amichund Chuturgoon, Terisha Ghazi, Kgothatso Eugene Machaba

**Affiliations:** 1 School of Laboratory Medicine and Medical Sciences, University of KwaZulu-Natal, Durban 4041, South Africa; 2 College of Health Sciences, University of KwaZulu-Natal, Durban 4041, South Africa

**Keywords:** diabetes mellitus, epigenetics, epi-drugs, DNA methylation, histone modifications

## Abstract

Type 2 Diabetes Mellitus (T2DM) is increasingly affecting individuals across various age groups due to inadequate insulin action and secretion. It has become the leading cause of mortality worldwide, with an estimated 9.3% of the global population currently affected. Recent epigenetic studies have shown that variations such as DNA methylation and histone modifications are implicated in the development of T2DM. However, epigenetically related conditions are known to be reversible, which could potentially pave the way for predicting and treating T2DM. This has led to the development of epigenetic modifier drugs, including histone deacetylase inhibitors (HDACi), histone acetyltransferase inhibitors (HATi), protein arginine methyltransferase inhibitors (PRMTi), DNA methyltransferase inhibitors (DNMTi), histone demethylating inhibitors (HDMi), and sirtuin-activating compounds (STAC). A major challenge with these epigenetic drugs is that only a few have been approved for treating metabolic diseases due to their potential to negatively impact off-target genes. The low specificity of these drugs can lead to side effects and increased toxicity, contributing to complex diseases such as cancer. Hence, gaining a comprehensive understanding of the epigenetic mechanisms underlying metabolic diseases can provide new insights and strategies for preventing, diagnosing, and treating metabolic disorders, such as T2DM. This review summarizes the epigenetic variations in T2DM, pharmaco-epigenetics, and the challenges surrounding epigenetics. This provides basic insight into the discovery of novel drug targets, which can lead to the development of epigenetic therapies for T2DM. Hence, the reversible nature of epigenetic variations retains hope for future novel strategies to combat T2DM.

## 1. Introduction

People of all ages are increasingly affected by diabetes mellitus (DM) due to insufficient insulin action and secretion [1]. As a result, diabetes has become a leading cause of mortality worldwide, currently impacting an estimated 9.3% of the global population, which amounts to around 734 million individuals [2]. The prevalence of DM is expected to increase to 822 million people (10.4%) by 2040 [3]. The anticipated increase is expected to be more pronounced in developing regions, such as Africa (from 16 million to 41 million; a 156% increase), the Middle East and North Africa (from 39 million to 82 million; a 110% increase), South and East Asia (from 82 million to 151 million; an 84% increase), and South and Central America (from 26 million to 42 million; a 62% increase). In contrast, Europe, North America, and the Western Pacific are projected to experience smaller percentage increases [4].

It is essential to highlight that DM mostly results from insufficient insulin synthesis or impaired insulin sensitivity. Hence, it can be categorized into various forms, including type 1 diabetes mellitus (T1DM) and type 2 diabetes mellitus (T2DM) [5]. T1DM is typically caused by the impairment of insulin production, resulting from the disruption of pancreatic β-cells as a consequence of T-cell-mediated autoimmunity [6], while T2DM is the predominant type of diabetes, characterized by insulin resistance (IR). Research has demonstrated that numerous factors significantly contribute to IR or insulin underutilization. These factors include genetics, environment, obesity, sedentary lifestyle, insufficient physical activity, smoking, high intake of alcoholic beverages, dyslipidemia, decreased β-cell sensitivity, hyperinsulinemia, and increased glucagon activity [7]. Hence, the sudden and swift changes in urbanization and diet are the primary reasons for the above projections [8]. In recent decades, diabetes has been increasingly affecting low- and middle-income countries at a faster rate than high-income nations [9]. South Africa has recently been confirmed to have a prevalence rate of 15.56% pre-diabetes [10], with countries such as Brazil having a prevalence of 9.2% of T2DM, spanning from 6.3% in the North to 12.8% in the Southeast [11].

**Figure 1:**
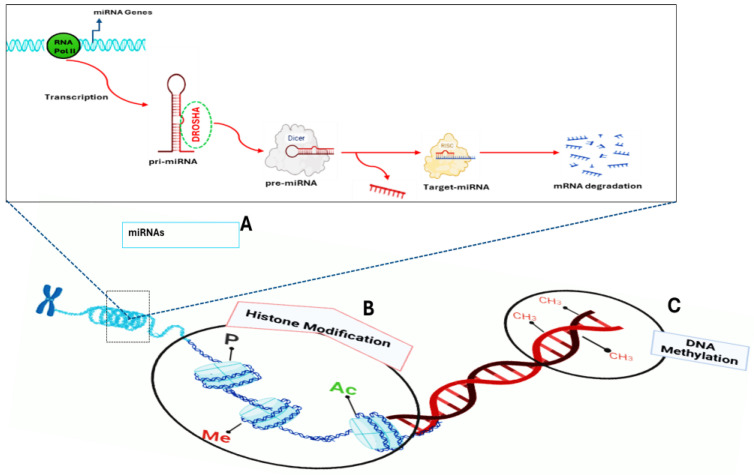
The diagrammatic illustration of epigenetic modifications, which include DNA methylation, histone modifications, and miRNAs. (**A**) Mechanism of miRNA synthesis. The enzyme called RNA polymerase II transcribes miRNA genes into pri-miRNAs, which are then cleaved by Drosha and DGCR8 into a hairpin structure called pre-miRNAs. The pre-miRNAs are then transported into the cytoplasm by Exportin-5, where Dicer cleaves pre-miRNAs into double-stranded RNAs. One strand is incorporated into the RNA-induced silencing complex (RISC) to guide mRNA targeting, while the other is degraded. (**B**) The core histones undergo histone modifications, which include histone methylation (me), where methyl groups are added to histone proteins, acetylation (Ac), where an acetyl group is added to histone proteins, and phosphorylation (P), where a phosphate group is added to specific amino acids within the tail of histone proteins. (**C**) DNA methylation, whereby DNMT enzymes add a methyl group to DNA. Source: Adapted from Desiderio et al. [15]. Created with BioRender.com.

Research has shown that the rise in T2DM prevalence is strongly associated with the worldwide increase in obesity since it affects both insulin and beta cell function [12]. Therefore, lifestyle choices can potentially induce reversible changes in gene expression without modifying the DNA sequence, a phenomenon known as epigenetics. [13]. Epigenetics is generally described as heritable changes in gene activity or expression that occur without altering the DNA nucleotide sequence. These changes can be passed on when cells divide and are often influenced by environmental factors [14]. Epigenetic regulation is primarily controlled by three key mechanisms: DNA methylation, histone modifications, and microRNA (miRNA)-mediated regulation, as illustrated in Figure 1 [13].

Among these, DNA methylation involves the addition of a methyl group to the DNA molecule, typically at cytosine–phosphate–guanine (CpG) sites, catalyzed by DNA methyltransferase (DNMT) enzymes [13]. Recent studies have suggested that alterations in DNA methylation patterns are implicated in the pathogenesis of obesity and T2DM [14]. Notably, specific DNA methylation sites associated with obesity have also been shown to predict the risk of developing T2DM [16]. For instance, in subcutaneous adipose tissue (SAT), researchers have identified six significant CpG sites within the *KCNQ1* gene that exhibit reduced methylation levels in individuals who have undergone weight loss [16]. Additional findings by Benton et al. [17] revealed differences in DNA methylation levels within SAT, particularly involving the DNA methyltransferase enzymes DNMT3A and DNMT3L. Notably, these enzymes exhibited decreased promoter methylation following weight loss. Furthermore, the gene encoding the MBD4 protein, which normally binds to methylated cytosines, showed reduced levels of DNA methylation after weight loss [17]. The above observed alterations in DNA methylation associated with weight loss and gain provide evidence that certain epigenetic modifications are stable over time, whereas others are dynamic and responsive to various environmental and physiological factors, including short- and long-term exposure to medications, dietary changes, physical activity, psychological stress, and the aging process [18].

This has led to the development of epigenetic drugs and epigenetic programs, which can effectively reverse epigenetic and transcriptional aberrations present in various diseases [19]. Nevertheless, the primary obstacle to epigenetic therapy is that its advantages are outweighed by its adverse effects [20]. Consequently, epigenetic drugs have encountered obstacles associated with disease treatment, such as (i) a lack of selectivity, (ii) restricted therapeutic efficacy, and (iii) inadequate safety [21]. Among the challenges associated with epigenetic drugs, the most prominent is their lack of specificity, which can lead to unintended effects on undesired genes [22]. For example, epigenetic drugs such as DNA methyltransferase inhibitors (DNMTi) cytidine analogs are known to be unstable and non-specific to their target; hence, there is a need to develop more precise and selective DNMTi [21].

Therefore, it becomes essential to investigate the epigenetic mechanisms that occur in response to epigenetic therapies and epigenetic variation in DM, particularly T2DM, and to gain insights into the challenges surrounding epigenetics. This review aimed to summarize the epigenetic changes that occur due to epigenetic therapies and understand the pharmacogenetic interaction. This would assist in understanding the underlying genes that get silenced and activated through epigenetic therapy. This could serve as a starting point for developing therapeutic derivatives against T2DM and obesity with minimal side effects.

## 2. Epigenetic Variation in T2DM, Drug Treatment, and Drug Response

Epigenetics is one of the most rapidly advancing fields in biology. It encompasses changes in gene expression that arise from chemical modifications of DNA and its associated proteins, without altering the underlying DNA sequence [23]. The regulation of gene expression by epigenetics has recently been associated with various metabolic diseases, including non-alcoholic fatty liver disease (NAFLD), diabetes, obesity, hyperthyroidism, gout, hypothyroidism, and osteoporosis [24]. Epigenetic modifications have been implicated in the pathogenesis of the aforementioned diseases, as epigenetic regulation is fundamentally associated with various biological processes, including cell replication, adhesion, and differentiation [25]. Consequently, several epigenetic alterations are increasingly recognized as potential biomarkers for metabolic disorders [26]. Therefore, understanding epigenetic alterations becomes crucial to elucidate the fundamental processes behind the effects of environment and lifestyle on health and disease development [23]. Notably, lifestyle factors significantly contribute to the risk of major chronic diseases such as obesity, T2DM, cancer, and cardiovascular disease [27].

It is essential to emphasize that epigenetic modifications, including histone modifications, DNA methylation, and non-coding RNAs (ncRNAs), are potentially reversible and can have enduring effects on gene expression [28]. This phenomenon may potentially provide new therapeutic pathways for treating diseases by utilizing epigenetic modulators [23]. Furthermore, epigenetics is a helpful tool in understanding the pathogenesis of diseases and providing biomarkers for disease diagnosis and risk classification. The primary limitation of epigenetics is that epigenetic interventions or therapies may potentially cause unexpected adverse reactions, including cancer, abnormalities, and adverse drug reactions [23]. Therefore, significant research is needed to develop safe and effective therapies that improve health and mitigate the risks associated with epigenetics. Hence, epigenetic codes such as DNA methylation, histone modifications, and ncRNAs (Figure 1) have been well-studied [29].

Interestingly, epigenetics has been recognized as one of the medical research areas that promises to influence disease development and management by individuals [30]. Therefore, primary interest has been in understanding various epigenetic processes implicated in diseases such as T2DM and obesity [31,32]. Previous authors have highlighted that even non-genetic factors, such as nutrition, exercise, etc., can alter epigenetic signatures, thereby modifying gene activity [33]. Beyond dietary factors, several non-nutritional risk factors have been associated with obesity, including oxidative stress, hyperglycemia, inflammation, hypoxia, and endocrine disruptors. These factors are known to induce epigenetic alterations, thereby influencing adipogenesis and insulin sensitivity [32].

Researchers have indicated that a healthy diet can beneficially influence the epigenetic profile of individuals. Hence, previous data have indicated that individuals who are non-diabetic with average body weight have a distinct epigenetic profile when compared to obese and diabetic individuals [34]. A high-fat diet (HFD) has been observed to disrupt the epigenetic profile in various ways, including the manipulation of energy homeostasis, disruption of hormone secretion, alteration in signalling processes, and the induction of inflammation [35]. Consequently, an unbalanced diet during pregnancy affects the fetal and neonatal microbiome, causing certain epigenetic signatures that may lead to obesity and T2DM later in life [36]. A balanced diet reduces the risk of obesity and T2DM by modification of DNA methylation patterns [37]. A study conducted by Parrillo et al. [38] demonstrated that when a mouse is fed an HFD, it leads to hypermethylation in the promoter region of the Hoxa5 gene within visceral adipose tissue. However, upon substituting the HFD with a regular chow diet, the Hoxa5 gene methylation returned to normal levels.

### 2.1. Epigenetic Variation in T2DM

Epigenetic variation influences gene expression and protein synthesis [39]; hence, it can result from a particular disease or be the cause of a particular disease [40]. Thus, previous researchers have indicated that epigenetic variation may cause complex metabolic diseases, including T2DM [41]. It should be noted that there are numerous environmental factors that can influence epigenetic variation through environment–gene interactions, highlighting the epigenome’s responsiveness to lifestyle-related changes [42]. For example, de Castro Barbosa et al. [43] reported that a HFD induced changes in the mouse epigenome; furthermore, an unbalanced diet during postnatal life was observed to cause epigenetic reprogramming and was associated with an increase in T2DM and obesity [44]. Other researchers also showed that T2DM and obesity are associated with DNA methylation changes [42].

Generally, DNA methylation and histone modifications are considered the primary epigenetic mechanisms contributing to metabolic disorders [45]. Nevertheless, additional mechanisms, including chromatin remodeling and ncRNAs, have also been identified as significant epigenetic regulators involved in various metabolic disorders [24].

#### 2.1.1. DNA Methylation

The methylation of DNA has been the most well-researched epigenetic mechanism. It has been investigated in numerous organisms and linked to various biological processes such as gene regulation, genome organization, reproduction, disease development, and ageing [46]. DNA methylation has been recognized as a type of modification whereby the environment possibly interacts with the genome. Hence, evidence indicates that the modifications in DNA methylation result in T1DM and T2DM [47]. This is likely due to DNA methylation being a crucial biochemical mechanism for modulating DNA activity.

**Figure 2:**
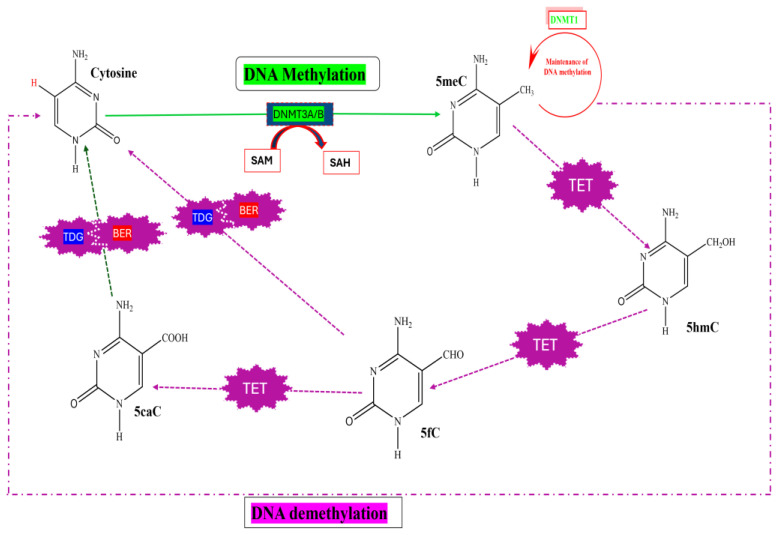
The DNA methylation and demethylation mechanism. cytosine (C); S-adenosylmethionine (SAM); S-adenosylhomocysteine (SAH); 5-methylcytosine (5meC); 5-carboxylcytosine (5caC); 5-formylcytosine (5fC); 5-hydroxymethylcytosine (5hmC); DNA methyltransferase (DNMT); ten eleven translocation enzyme (TET); thymine-DNA-glycosylase (TDG); base excision repair (BER). Created with ChemDraw 23.0 professional software.

DNA methylation involves the covalent addition of a methyl group to the fifth carbon of the cytosine ring, resulting in the formation of 5-methylcytosine (5meC), or alternatively, the methylation of adenine at the sixth nitrogen of the purine ring. Specifically, DNA methyltransferase (DNMT) enzymes catalyze the transfer of a methyl group (CH_3_) from the methyl donor S-adenosyl-L-methionine (SAM) to the cytosine residue, as illustrated in Figure 2 [48]. DNA methylation predominantly occurs in gene promoter regions, where it typically represses gene transcription. This epigenetic alteration is heritable through cell differentiation and has been linked to the onset and progression of various diseases, including T2DM. The methylation process comprises three main mechanisms: de novo methylation, recognition of 5meC, and both active and passive demethylation.

It has been initially established that de novo DNA methylation is mediated by the enzymes DNMT3A and DNMT3B, which act predominantly at symmetrical CpG sites, and the methylation marks are subsequently maintained by DNMT1 during DNA replication, as illustrated in Figure 2. The catalytic activity of DNMT3A and DNMT3B, along with the involvement of the regulatory cofactor DNMT3L, is essential for this process [49]. During this reaction, CH_3_ groups are transferred to the cytosine residues of previously unmethylated DNA strands, thereby establishing stable epigenetic marks [50]. Furthermore, DNMT1 then maintains DNA methylation by restoring the methylation status on newly synthesized DNA strands. It specifically recognises hemi-methylated CpG sites, where the parental strand is methylated and the complementary daughter strand is unmethylated, and catalyzes the addition of a methyl group to the newly synthesized strand [51]. This function is facilitated by the interaction of DNMT1 with its key cofactor, ubiquitin-like with PHD and RING finger domains 1 (UHRF1), which detects hemi-methylated DNA and recruits DNMT1 to replication foci [52]. Thus, the above-mentioned multi-domain protein directs the DNMT1 enzymes to the hemi-methylated CpG regions [51,52].

DNA methylation is a fundamental epigenetic process through which 5meC modifies gene activity [53]. 5meC may influence or modify gene activity by physically inhibiting transcription factors from binding to gene promoters, which results in gene silencing. They can also modify gene activity by attracting methyl-CpG-binding proteins, which recognise 5meCs and trigger changes in the expression of genes [53]. It is also imperative to highlight that, in addition to CpG sites, there are regions of genes that have a high concentration of CpG sites, known as CpG islands (CGIs). These regions are considered transcriptional regulatory regions and are typically connected with the promoter region of multiple genes [54]. These regions are typically unmethylated and are therefore associated with active gene expression. However, when methylation occurs in these regions, it can lead to gene silencing [53]. As previously stated, 5meCs play a fundamental role in regulating and changing gene activity; nevertheless, 5meC can be reversed or changed back to its original cytosine through passive or active demethylation [55]. During passive demethylation, the maintenance of DNA methylation by DNMT1 gets interrupted [56] due to downregulation of DNMT1 expression during cell replication [45]. This results in either empty or hypomethylated CpG sites on the newly synthesized strand, while the parent strand retains its methylation status [56]. In contrast to passive demethylation, active demethylation is mediated by ten-eleven translocation (TET) dioxygenases through a series of enzymatic steps. TETs enzymatically oxidise 5meC to 5-hydroxymethylcytosine (5hmC), 5-formylcytosine (5fC), and 5-carboxylcytosine (5caC), as observed in Figure 2 [57]. Subsequently, 5fC and 5caC are excised through the action of thymine DNA glycosylase (TDG), ultimately resulting in the active removal of the methyl group [58]. DNA methylation and demethylation are involved in various mechanisms underlying tissue growth and development, primarily due to their dynamic occurrence within the same genomic regions [59]. Hence, it becomes essential to identify or characterise alterations in DNA methylation and demethylation for comprehending disease progression and development [60]. Since aberrant DNA methylation is associated with disease progression, maintaining a normal DNA methylation status is essential for the proper growth and development of organisms [61]. While DNA methylation is generally associated with gene silencing, DNA demethylation is indicative of gene activation [60].

For instance, previous research has shown that DNA methylation negatively affects gene expression in individuals with T2DM, particularly in skeletal muscle and pancreatic islet cells [62]. According to findings by Taneera et al. [63], glucagon-like peptide 1 receptor (GLP1R) expression in pancreatic islets is significantly reduced in patients with T2DM as well as in hyperglycemic rat models. Furthermore, Hall et al. [64] demonstrated that DNA methylation at CpG sites proximal to the transcription start site of the GLP1R promoter can negatively influence gene expression. Collectively, these findings suggest that CpG site methylation has a deleterious effect on GLP1R expression, ultimately contributing to reduced insulin levels and the pathogenesis of T2DM.

#### 2.1.2. Histone Modifications

Histones are proteins that bind and organize the DNA of eukaryotic cells. Hence, they are recognized as chromatin’s significant chief protein components [65]. These proteins serve a significant function in determining chromatin’s structure and regulating gene activity [65]. The histone proteins are said to be highly conserved with similar structural folds, of which only four histone classes (H2A, H2B, H3, and H4) are recognized as the core histone subunits [66], that bind to 147 base pairs of DNA. On the contrary, H1/H5 proteins attach to the DNA linker sequence [65]. The H1/H5 proteins serve as an interlinker between two units of nucleosomes, which promote stabilization and advanced packaging of chromatin within the nucleus [67]. The histone proteins undergo post-translational enzymatic modifications [68] via histone-modifying enzymes, namely histone acetyl transferases (HATs) and histone deacetylases (HDACs) [69]. As mentioned above, post-translational enzymatic modifications influence nucleosome dynamics, hence affecting DNA activity [69].

To name a few, there are numerous post-translational modifications, which include methylation (arginine and lysine), acetylation (lysine), ubiquitination (lysine), sumoylation (lysine), phosphorylation (serine, tyrosine, and threonine), ADP-ribosylation, and glycosylation that occur at the N-terminal tails of histones. These modifications change the functional properties and structure of chromatin; for example, they disrupt electrostatic interactions between histones and DNA, which affects chromatin compactness [70]. To be precise, there are two mechanisms through which the chromatin structure and function get affected, namely, (a) a decrease in affinity between histones and DNA via changing the electrostatic charges of amino acids around the histone proteins and (b) the formation of a surface that interacts with particular protein complexes and binds to protein interaction factors to control transcription activity [71]. Out of the mentioned post-translational modifications, acetylation, methylation, ubiquitination, and phosphorylation (Table 1) are the major post-translational modifications [72]. For instance, acetylation and methylation are considered to be the most meticulously researched histone modifications associated with diabetes [73].

However, it is important to emphasize that the significance of alterations largely depends on the type, extent, and genomic location of the modification [74]. Hence, numerous histone modifications can cause the chromatin to be euchromatin (active) or heterochromatin (inactive), thus affecting repair, replication, and recombination of DNA, and also altering gene transcription together with alternative splicing [75]. The modifications, such as histone acetylation and histone phosphorylation, are linked with the activation of transcription. In contrast, histone methylation is usually linked with either activation of transcription or repression, which is determined by the level and site of methylation [76]. Since histones have a high amount of basic amino acid (lysine and arginine) residues, they are positively charged proteins [77] with electrostatic DNA interactions [78]. Histone acetylation arises through the addition of an acetyl group from acetyl-CoA to the ε-amino group of lysine to form the ε-N-acetyllysine. This results in the neutralization of the positive charge on the lysine residue. The neutralization of the positive charges on the residues of lysine weakens the bond between the histone and DNA, causing histones to drift away from DNA and activate transcription [79]. The histone acetylation modification is catalyzed by HATs. However, the process can be reversed through the removal of the acetyl group by the action of HDACs [79]. HATs can be grouped into numerous families depending on their homology sequence (Table 1) [79,80].

**Table 1:** Histone modifiers (writers and erasers) and their associated modifications.

Type of Modification	Histone Proteins	Amino Acid Residues	Associated Biological Effect	Writers	Erasers	References
Ac	H2A	K5, k9, k13, k15, k36, k74, k95, k118, k127, k129	Activation of a gene	p300/KAT3B, Tip60/KAT5, MYST2/KAT7	HDAC5, SIRTs (SirT1, SirT2, SirT6)	[79,81]
	H2B	K5, k11, k12, k15, k16, k20	Activation of a gene	CBP/KAT3, Ap300/KAT3B	-	[82,83]
	H3	K4, K14, K18, K23, K36	Activation of a gene	-	-	[79]
		K9, K27	Activation of a gene	MOF, p300, PCAF, TIP60	HDAC	[79]
	H4	K5, K8, K16	Activation of a gene	p300/KAT3B, Tip60/KAT5, MYST2/KAT7, ELP3	-	[79]
	H3	K4	Activation of a gene	ASH1L, MLL1-4, SET7/9, SETD2A-B, SMYD	JARID2, KDM1A-B, KDM2B, KDM5A-D, NO66	[79,84]
		K9	Repression of a gene	GLP, G9a, SETDB1-2, SUV39H1-2	JHDM1D, KDM1A, KDM3A-B, KDM4A-E, KDM7, PHF8	[79]
Me		K27	Repression of a gene	EZH 1-2, PRC2	UTX, UTY, JMJD3, KDM7, PHF8	[79]
		K36	Activation of a gene	ASH1L, NSD1-3, SMYD, SET2	KDM2A-B, KDM4A-E, NO66	[85]
		K79	Activation of a gene	DOT1L	PHF8	[79]
	H4	K2O	Repression of a gene	SET8, SUV4-20H1	PHF8, PHF2	[79,84]
		R3	Activation of a gene	PRMT1, PRMT3, PRMT5	JHDM1D/KDM7A, PHF8	[79]
P	H2A	S1	Mitosis	MSK1, PKC	-	[79]
		S16	eGF signaling	rSK2	-	[86]
		T120	Mitosis, gene activation	BUB1, NHK1, VprBP	-	[86]
	H2B	S32	eGF signaling	rSK2	-	[86]
		S14	Apoptosis	MST1	-	[79]
		S36	Transcription	AMPK	-	[86]
	H3	S14	Apoptosis	MST1	-	[79]
		S10	Mitosis, DNA repair	MSK1&2, AuroraA	PP1	[87]
		T6	Activation	PKCβ	-	[88]
	H4	T11	Mitosis, DNA repair	DLK/ZIP, PRK1	-	[88]
		S1	Mitosis, gene activation	CKII, ScCK1	-	[88]
Ub	H2A	K119	Repression of a gene	BMI/RING1A	-	[89]
	H2B	K120	Activation of a gene	RNF20-RNF40	-	[90]
	H3	K23	Maintenance of DNA methylation	UHRF1	-	[79]
Ser	H3	Q5	Activation of a gene	TGM2	-	[79]
La	H3	K18	Activation of a gene	p300	-	[91]
	H4	K12	Activation of a gene	p300	-	[91]
Cr	H3	K9	DNA repair	p300, GCN5, MOF	HDAC1	[79]
		K18	Activation of a gene	p300, GCN5, MOF	-	[79]
		K27	Gene activation	GCN5	-	[79]

Acetylation (Ac), methylation (Me), phosphorylation (P), ubiquitination (Ub), serotonylation (Ser), lactylation (La), crotonylation (Cr), unknown (-).

Similarly to acetylation, histone phosphorylation alters the charge of histone proteins by targeting the N-terminal tails, where amino acid residues such as serine, threonine, and tyrosine undergo phosphorylation [86,92]. The mechanism of histone phosphorylation is through the synergistic activity of enzymes, such as phosphatases and kinases, that create or eliminate the modification [93]. Specifically, the phosphate groups from adenosine triphosphate (ATP) are transferred into hydroxyl groups of serine, threonine, and tyrosine residues [70]. The negatively charged phosphate groups, which are added, repel the negatively charged DNA, which activates gene transcription in a similar way as in histone acetylation [94]. Enzymes such as protein kinases catalyze the process of adding a phosphate group; conversely, phosphatase enzymes are responsible for the removal of phosphate groups, thereby reversing the effects of phosphorylation [95]. The phosphorylation of histones has previously been associated with the expression of genes involved in cell proliferation and cell cycle-related processes, including DNA repair [96]. Hence, it has been connected to endothelial dysfunction, diabetic kidney disease, and cardiometabolic disorders such as adipogenesis [97]. For example, the phosphorylation of serine 10 in the tails of histone H3 has been observed to mediate the function of cells in diabetes and contribute to the activation of genes in diabetic kidney disease [98].

In contrast to the aforementioned modifications, histone methylation occurs on basic amino acid residues, primarily arginine, lysine, and histidine [99], and can lead to either transcriptional activation or repression, depending on the specific residue methylated and the degree of methylation [76]. Unlike histone phosphorylation and acetylation, histone methylation does not alter the charge of the histone protein. Therefore, histone methylation does not disrupt the interaction between histones and DNA; rather, it serves as an informational marker, storing regulatory cues [100]. The process of histone methylation can be reversibly modulated through the action of enzymes, such as histone methyltransferases (HMTs) and histone demethylases (HDMs), that function to either add or delete the methyl groups from amino acid residues. For example, HMTs transfer the methyl group from SAM to arginine, lysine, and histidine residues, respectively [101]. The methyl group can be removed from arginine, histidine, and lysine residues by HDMs, which maintain histone methylation homeostasis [102]. It is important to note that histone methylation can occur in multiple forms. For lysine residues at the ε-amino group, methylation may present as mono- (me1), di- (me2), or tri-methylation (me3). In contrast, arginine residues may undergo mono-methylation (me1) or di-methylation, which can be either symmetrical (me2s) or asymmetrical (me2a) [103]. Therefore, the above can result in either transcriptional activation or transcriptional repression of a gene, depending on the extent of methylation and different residues modified [76]. For instance, mono-methylation of histones at the lysine residues is usually linked with repression of the gene. In contrast, the di- or tri-methylation of lysine potentially enhances gene transcription (H3K4me3), while inducing gene silencing on H3K9me3/me2 [104] or gene repression when it is linked to hypermethylation in CpG islands of promoter regions (H3K27me3) [105]. Hence, previous research indicated that the development of T2DM was related to a high level of H3K4me1 and H3K9me2, while the H3K9ac and H3K23ac levels were reduced [106].

### 2.2. Non-Epigenetic Drug Responsiveness Is Affected by Epigenetic Variation

Epigenetic variation has been observed in close proximity to specific gene regulators and genes associated with drug metabolism [107]; therefore, such variability may significantly influence individual responses to pharmacological treatments [108]. Previous studies have stipulated that epigenetic changes could drive drug resistance accumulation [109]. Hence, other authors have also highlighted that epigenetic alterations affect non-epigenetic drug response. For instance, Jacquemont et al. [110] stipulated that there is a correlation between DNA methylation patterns and a varied reaction to a mGluR5 antagonist medication in patients with fragile X syndrome, therefore mitigating certain symptoms of the disorder. Furthermore, epigenetic biomarkers may open a new avenue for better drug response prediction. Hence, epigenome biomarkers play a role in monitoring the drug treatment efficacy [111].

The epigenetic variations also have a significant role in epigenetic imprinting, such as drug metabolism. In fact, research by Nakajima et al. [112] and Li et al. [113] has indicated that methylation of DNA at the promoter region of the gene potentially modulates the expression of cytochrome (CYP) enzymes, namely CYP1A1, CYP1A2, CYP1B1, CYP2C19, CYP2D6, CYP3A4, and CYP3A5. Thus, the expression of the CPY enzyme is decreased due to hypermethylation of the promoter gene, particularly for drugs metabolized by CPY enzymes. Therefore, the decrease in enzyme expression causes a high concentration of drug within the site of action and increases the drug’s duration [114]. Various researchers have also proved the above phenomenon. Hence, they have stipulated that a level of methylation in a gene’s promoter regions causes a reduced expression of certain genes [115]. However, it should also be emphasized that methylation of the transcribed regions results in variable impacts on gene expression. Hence, DNA methylation patterns affect drug response since DNA methylation regulates the expression of genes [116]. For instance, research conducted by García-Calzón et al. [117] established that drug-naïve individuals who have been newly diagnosed with T2DM exhibited significantly elevated DNA methylation levels at 11 CpG sites in their blood. This was, therefore, associated with a higher risk of non-responsiveness to metformin, while a further methylation of four additional CpG sites was linked with a higher risk of metformin intolerance.

### 2.3. Key Epigenetic Biomarkers Predict Diagnosis and Prognosis

Further research into the epigenetic mechanisms underlying T2DM is essential to elucidate its pathogenesis and identify potential therapeutic targets, as the disease has become increasingly complex due to the interplay between genetic predisposition and environmental factors [102]. Hence, research that will clarify how epigenetics might be used as relevant biomarkers for prognosis, risk assessment, and diagnosis is currently necessary [118].

Emerging evidence from Raciti et al. [119] suggests that human epigenetic modifications may serve as potential biomarkers for predicting lifelong risk of developing obesity and T2DM, even before the onset of clinical symptoms. The above phenomenon indicates that there is a need for biomarkers that could accurately predict gestational diabetes (GDM) and its related adverse outcomes, which can be at perinatal or later (offspring and maternal) in life. This intervention can benefit both mothers and offspring by reducing the future risk of obesity and T2DM due to early interventions (lifestyle or pharmaceutical) [120]. The discovery of novel translational epigenetic biomarkers has helped identify people who are more likely to be at risk of developing T2DM, enhancing prognostic and diagnostic techniques and forecasting treatment and lifestyle interventions [121]. Nonetheless, technology that is capable of identifying novel biomarkers is still under development and scarce, and there is still a need to enhance the scope and calibre of available tools [121]. Furthermore, other researchers have also stipulated that identifying epigenetic biomarkers for metabolic diseases is still scarce [122].

It should be noted that epigenetic modifications, such as variations in DNA methylation, have been associated with numerous diseases, which include T2DM [123]. For example, Yang et al. [124] have previously indicated that an increase in DNA methylation resulted in the decreased expression of genes such as the insulin gene and PDX1, which is a crucial aspect for the function and development of β-cells in pancreatic islets of diabetic patients.

DNA methylation is the potential ideal epigenetic biomarker since it is well understood and is highly useful in the analysis of human disease because it has proven to be stable over time and is simple to measure [125]. Notably, a study by María Martín-Núñez et al. [126] associated the risk of impaired glucose metabolism with reduced LINE-1 methylation levels, reflecting alterations in global DNA methylation. Thus, the above-mentioned biomarker is regarded as a risk factor for T2DM and associated metabolic diseases [127]. Previous research by Toperoff et al. [128] has also associated DNA methylation with T2DM; for example, the study indicated that DNA hypomethylation in specified sites for young individuals later resulted in the development of T2DM. Furthermore, Nilsson et al. [129] compared adipose tissue-specific CpG sites of healthy controls and individuals with T2DM. Hence, it was established that they exhibit differential DNA methylation in various genes related to T2DM (PPARG, IRS1, and TCF7L2). In addition to DNA methylation, other epigenetic biomarkers such as microRNAs and post-translational modifications (PTMs) of histones have also been shown to remain stable in various biological fluids (including plasma, serum, urine, saliva, semen, and vaginal secretions) [130], as well as in primary tissue samples, such as frozen or fresh tissue, dried blood spots (Guthrie cards), and formalin-fixed paraffin-embedded (FFPE) tissues [131]. However, it is important to emphasize that primary critical biomarkers facilitate the prediction of future health outcomes, which aligns with the core objectives of current public health strategies [132]. Furthermore, according to García-Giménez et al. [133], an ideal biomarker could be found in readily available samples such as blood and should indicate modifications in tissues that are slightly reachable. Generally, biomarkers are essential to health care because they play a significant role in disease prognosis and diagnosis, response to treatment, and personalised medicine. They provide early disease detection and appropriate intervention, thus improving patient conditions at a reduced cost [134].

### 2.4. Epigenetic Strategies for Diabetes

In efforts to combat obesity and diabetes, recent research has focused on modulating disease-specific gene expression. This led to the development of epigenetic modifier drugs, such as histone acetyltransferase inhibitors (HATi), DNMTi, histone deacetylase inhibitors (HDACi), sirtuin-activating compounds (STACs), and histone demethylase inhibitors (HDMi) [135]. These epigenetic modifiers act as inhibitors or activators of histone-modifying enzymes and DNA methyltransferases, thereby influencing the interpretation of resulting chromatin alterations by epigenetic readers [136]. These epigenetic drugs target a vast network of biological molecules involved in signaling and metabolic pathways [136]. HDACi are beneficial in T2DM and obese individuals. It has been observed that the HDACi sodium phenylbutyrate can ameliorate IR and β-cell dysfunction, which are caused by elevated levels of free fatty acids in obese individuals; this is achieved through the reduction in inflammatory events related to endoplasmic reticulum stress [137]. Research conducted by Lewis et al. [138] demonstrated that the oral treatment with HDACi ITF2357 enhances β-cell viability and augments insulin secretion, while diminishing the synthesis and activity of pro-inflammatory chemokines.

One of the popular drugs for diabetes, metformin, causes a protective effect in vascular cells via glucose-lowering and pleiotropic effects that include DNA methylation. Metformin treatment typically results in a combination of hypo-, hyper-, and non-differential methylation patterns across CpG regions [139]. Furthermore, there is a class of therapeutic drugs called glucagon-like peptide 1 (GLP-1) agonists and GLP-1 receptor agonists, which prevent or inhibit complications from diabetes by modulation of epigenetic factors [140]. This class of therapeutic agents prevents hyperglycemia-induced DNA demethylation, which alters the promoter regions of *SOD2* and *NF-κB*, by reducing the binding of ten-eleven translocation methylcytosine dioxygenase 2 (TET2) to these sites [141]. Therefore, they cause a decrease in the expression of NF-kB target and NF-kB activation genes (TNF, IL-6, VCAM1), concurrently increasing SOD2 expression within the inflamed vascular cells [141]. Since the discovery of GLP-1, the treatment of diabetes has been redirected [142] to GLP-1 because it has numerous advantages, including suppression of appetite, delay of gastric release, decreased blood glucose levels, and further promotion of insulin secretion [143]. This phenomenon has resulted in the development of drugs such as Ozempic, which closely mimics GLP-1 and induces weight loss by delaying gastric emptying, enhances insulin sensitivity, and reduces hepatic fat accumulation [144]. It is noteworthy that therapeutic techniques aimed at epigenetic modifications in T2DM are presently in the preliminary stages; however, several promising approaches currently exist [145]. The epigenetic-based therapy is a promising treatment option since it can directly control gene expression at the pre-transcriptional level, hence addressing the root cause of gene dysregulation [20]. Thus, various compounds with epigenetic activity are currently under clinical study [145].

## 3. Combining Existing Pharmaco-Epigenetic Data to Explore Pharmaco-Epigenetic Correlations

The advancement in epigenetics has influenced pharmacology to develop a new speciality, namely, pharmaco-epigenetics. Pharmaco-epigenetics deals with the study of heritable non-genetic variations in response to drugs and drug toxicity, together with their fundamental mechanisms [146]. The present review seeks to boost the understanding and knowledge of how drugs work and their adverse reactions. Such knowledge will help identify new drug targets, which will help create epigenetic therapies that can cure epigenetic abnormalities in various diseases [147]. The drug response has undoubtedly been affected by various epigenetic factors, which have also been proposed to influence the expression of nuclear receptors, drug transporters, and drug-metabolizing enzymes, which eventually regulate gene expression [148]. As a result, it has been proposed that the following methods could be used to examine the correlation between epigenetics and drug responsiveness, using information that is currently available: (a) identifying regions of epigenetic variance, (b) identifying important epigenetic biomarkers, and (c) connecting the biomarkers with phenotypes associated with drug response [108].

The research conducted by García-Calzón et al. [117] evaluated whether methylation in blood taken before treatment may distinguish T2DM in individuals who were metformin-tolerant compared to those who were metformin-intolerant. The aforementioned study examined DNA methylation at approximately 850,000 sites in blood samples from metformin intolerance discovery and replication cohorts, which included treatment-naïve patients with T2DM. Intolerable side effects were linked to 12,579 regions of DNA methylation in the discovery cohort. Their analysis revealed that most sites had greater methylation (7865 CpGs) in patients who were metformin-intolerant as opposed to those who were metformin-tolerant.

García-Calzón et al. [117] further investigated the response of blood sugar to metformin, where they silenced the SEPT11 and CST1 genes, which are located near CpG sites associated with metformin response, in recently diagnosed individuals with T2DM. It was discovered that a deficiency of SEPT11 in HepG2 cells resulted in lower expression of SLC47A1, which could potentially cause reduced efflux/elimination of substances, thereby resulting in higher metformin concentrations within hepatocytes. This is therefore linked to a greater pharmacological response [149]. Metformin has been the first-line drug for treating T2DM [117]. However, it has recently been determined that other epigenetic drugs are implicated in metabolic diseases [24]. Remarkably, the U.S. FDA has not yet approved these epigenetic drugs for the treatment of metabolic illnesses; instead, they have only approved them for other conditions [24]. For instance, DNMTi drugs, including azacytidine, decitabine, and guadecitabine, have the potential to treat metabolic diseases but haven’t received approval for the treatment of metabolic illnesses. Instead, they are approved for other diseases. It is known that hydralazine may protect diabetic kidney disease (DKD) patients by inhibiting reactive oxygen species (ROS) through inhibiting xanthine oxidase (XO), consequently stimulating Nrf2-mediated heme oxygenase 1 (HO-1) [150]. It is noteworthy that a low dose of hydralazine reduces obesity-associated kidney disease, which can be reversed by the mechanism that involves reducing obesity-induced methylation levels within the whole kidney [151]. Another DNMTi drug called procainamide is known to partially inhibit the DNMT1 enzyme, and it protects against diabetes in mice with a deficiency in the unconventional prefoldin RPB5 interactor (URI), by decreasing PDX1 methylation levels and restoring PDX1 expression [152].

Besides the DNMTi drugs, other medications related to histone modification have been developed. These drugs include HDACi, HATi, and STAC, which have undergone different phases of approval, as observed in Table 2 [24]. Numerous HDACi have received approval from the U.S. FDA, including vorinostat, valproic acid, and sodium phenylbutyrate, to name a few [24].

HDACi have played a major role in treating diabetes by inhibiting hyperglycemia and reversing diabetes within the newly diagnosed non-obese diabetic mice [153]. Out of the HDACi mentioned above, valproic acid (VPA) has been known as an antiepileptic drug, which has also been indicated to be involved in diabetes treatment. Hence, the VPA has been observed to have numerous anti-diabetic mechanisms, which include reduction in H3 acetylation in the pancreas and, therefore, suppression of the apoptosis of β cells [154]. Additionally, the VPA promotes Treg differentiation by upregulating the expression of H3 acetylation and STAT5, which prevents autoimmune responses during islet transplant and aids in T1DM therapy [155]. It should also be highlighted that some other epigenetic drugs, such as HATi, have been well-researched for treating metabolic diseases. However, they have not received approval from the U.S. FDA [24]. These drugs include curcumin, which reduces the progression of DKD by increasing H3 histone acetylation and, thereby, reducing HSP-27 and p38 expression [156]. Besides the above-mentioned drugs, there are many other DNMT and HDAC inhibitors that are presently in preclinical phases or undergoing clinical trials, as observed in Table 2 [157].

However, it is crucial to emphasize that epigenetic therapy is emerging as a prospective treatment for a diverse array of diseases [158], despite the fact that the U.S. FDA has not yet approved any epigenetic drugs for the treatment of metabolic diseases [24]. For instance, certain HDACi have been identified as potential therapeutic agents for metabolic diseases, even though they have not yet been approved by the U.S. FDA. Consequently, drugs such as Givinostat (a lysine deacetylase inhibitor, KDACi) may prevent diabetes by protecting β cells from inflammatory harm [138]. 

Furthermore, it is acknowledged that epigenetic patents are increasingly progressing beyond fundamental laboratory techniques for detecting and modifying epigenetic changes, toward more advanced applications, including biological correlation studies, therapeutic interventions, and the archival of epigenetic data for future analysis. In contrast to patents that claim isolated DNA sequences, the majority of epigenetic patents are categorized as method-based claims [159].

The epigenetic patents are currently recognized to be in the midst of evolution from basic laboratory techniques for detecting and modifying epigenetic changes, to methods of using those techniques to observe biological correlations, make therapeutic interventions, and store epigenetic data for subsequent retrieval and analysis. Unlike DNA sequence patents that claim isolated DNA molecules, most epigenetic patents are method patents [159]. For example, in 2017, the H. Lee Moffitt Cancer Center and Research Institute (Tampa, FL, USA) filed patents in epigenetic targeting that describe compositions and methods for diagnosing, predicting, and selecting therapies for diseases such as cancer, obesity, and diabetes. These patents focus on histone phosphorylation at specific tyrosine residues, the kinases responsible for this modification, and the regulation of downstream genes affected by the phosphorylation [160].

**Table 2:** Various epigenetic drugs and their respective phases of clinical trials in the treatment of obesity and diabetes.

Drug Name/Type	Conditions	Status	NCT Number	Phase	References
STAC Resveratrol	T2DM	Completed	NCT01354977	Phase 2	[24]
	T2DM	Completed	NCT02549924	Phase 2	[24]
	Gestational Diabetes	Recruiting	NCT01997762	Phase 4	[161]
	T2DM	Completed	NCT01677611	Phase 1	[161]
	T2DM	Active,	NCT03762096	Phase 2	[162]
	T2DM	Recruiting	NCT01302639	N/A	[161]
	Obesity, insulin sensitivity, T2DM	Completed	NCT01412645	N/A	[135]
HATi Curcumin	T2DM; Dyslipidemias;	Recruiting	NCT05753436	Phase 2	[162]
	Hypertension				
	T1DM	Completed	NCT01646047	N/A	[163]
	Pre-diabetes; T2DM	Unknown	NCT01052025	Phase 4	[162]
	T2D, obesity	Unknown	NCT03542240	N/A	[24]
HDACi					
ValproicAcid	Diabetes	Completed	NCT00287352	Phase 1	[134]
ValproicAcid	Obesity	Unknown	NCT00298857	Phase 4	[24]
Sodium phenylbutyrate	Obese with insulin resistance	Completed	NCT00771901	N/A	[161]
DNMTi	T2DM	Completed	NCT00000620	Phase 3	[134]
Hydralazine	T2DM Hypertension	Recruiting	NCT02046395	Phase 4	[161]

Sirtuin-activating compounds (STAC), histone acetyltransferase inhibitors (HATi), histone deacetylase inhibitors (HDACi), DNA methyltransferase inhibitors (DNMTi), Clinical Trials.gov Identifier number (NCT), Type 2 Diabetes Mellitus (T2DM), Type 1 Diabetes Mellitus (T1DM), not available (N/A). Source: Adopted from Arguelles et al. [135].

## 4. Combining Existing Pharmaco-Epigenetic Data to Explore the DNA Methylation Profile of CYP Genes

Epigenetic drugs such as DNA methyltransferase inhibitors and histone deacetylase inhibitors have received significant attention, particularly in their application to the management of cancer [22] and diabetic retinopathy [164]. Hence, the above-mentioned drugs have been approved by the U.S. FDA for treating patients. However, the major concern is that these epigenetic drugs are non-specific in their action [165]. Such non-specificity in action affects genes that are not targets, thus resulting in negative effects such as carcinogenicity [22]. It should be noted that negative drug reactions (ADRs) are a primary contributor to mortality and morbidity within developed nations [166]. Therefore, research fields of pharmaco-epigenetics and toxico-epigenetics have aspired to improve knowledge of drug mechanisms and adverse drug responses. This would assist in the establishment of new targets for drugs, which would eventually lead to the development of epigenetic treatments that can cure epigenetic abnormalities within a wide range of illnesses [147].

The CYP family of proteins contribute significantly to drug metabolism processes, and they have been known to have inter- and intra-individual variability in activity and expression. This inter- and intra-individual variability of action is caused by both genetic and non-genetic factors [108]. Intra-individual variability is generally caused by genetic polymorphisms. However, epigenetic processes such as DNA methylation, histone modifications, microRNAs, and long non-coding RNAs also contribute to intra-individual variations [167]. For example, previous studies have discovered that DNA methylation controls the regulation of a drug-metabolizing enzyme (DME) within colon cancer cells [168]. The hypermethylation of CpG sites in the 5′-promoter regions of CYP1B1 and pregnane X receptor (PXR) genes resulted in the transcriptional upregulation of CYP1B1 and CYP3A4 genes due to demethylating agent or treatment [169]. Furthermore, DNA modification, namely 5hmC, has been re-established to have a significant role in controlling the expression of genes [170]. Hence, Ivanov et al. [170] confirmed the contribution of 5hmC DNA methylation in the expression and regulation of drug-metabolizing enzymes (DMEs) within the liver [170]. Furthermore, previous experimental evidence suggested that DNA methylation within gene promoter sites can turn off the expression of the CYP gene by preventing the binding of transcription factors to their binding sites [171]. Furthermore, consistent findings from other studies have shown that DNA methylation represses gene expression by obstructing transcription factor binding or by facilitating the recruitment of methyl-DNA-binding proteins, which subsequently attract histone deacetylases and promote chromatin condensation [172]. Thus, methylation of CpG islands plays a major role in the transcriptional silencing of many genes, which can be reactivated by demethylation [171]. Consequently, Okino et al. [173] showed that CYP1A1 silencing in the prostate cancer cell line LNCaP was caused by hypermethylation, but not in the noncancerous RWPE-1 cell line. Furthermore, it has been shown that certain factors, such as smoking, might affect the expression of CYP1A1. Hence, it was observed that for smokers, merely around 33% of heavy smokers, 71% of light smokers, and 98% of nonsmokers exhibited complete or partial methylation of CYP1A1 in their lung tissues. In addition, it was also reported that as early as one to seven days after stopping smoking, there was an increase in the methylation level, which may account for the smoking-related rise in CYP1A1 expression [174]. Other studies have also revealed that methylation of the CYP2R1 region lowers the secretion of plasma 25-hydroxyvitamin D [25(OH)D], which causes Vitamin D deficiency, leading to interruption in the insulin secretion, hence facilitating T2DM [175]. Smith et al. [108] have emphasised that it is important for scientists to understand the epigenetic landscape of the gene and also to know which genes are under regulation by genetic control; this will assist in better prioritising the gene sites to be pursued for biological experiments. This came after it was observed that most of the DNA methylation levels had been reported on CpG sites (e.g., 52% of DNA methylation levels were reported on ~420,000 tested). Hence, out of 10 evaluated CYP genes (CYP1A1, CYP2B6, CYP2C19, CYP2D6, CYP3A4, CYP3A5, and CYP4F2), seven of them have at least one DNA methylation site within their promoter region.

## 5. Challenges in Pharmaco-Epigenetics

Epigenetic drugs such as inhibitors of DNA methyltransferase and histone deacetylase have been well researched, especially in their management of cancer [22], and they have recently received approval from the US FDA. However, the major challenge with these epigenetic drugs is their non-specificity, which can affect unwanted targets for genes, thus causing negative effects such as carcinogenicity [22].

Therefore, epigenetic mechanisms/alterations have been observed to be linked with toxicological effects of drugs and chemicals or carcinogenicity [176], which potentially outweigh the benefits since epigenetic therapies have shown challenges of tolerability and dosing [20]. Current trials of epigenetic therapies have surprisingly demonstrated higher levels of toxicity than anticipated; this was possibly due to low specificity and the prevention of effectors due to the lack of cell type and genomic specificity. The above-observed results can drastically limit the utility of epi-drugs [20]. Compounds such as 5-azacitidine [174] and decitabine have recently gained approval by the FDA. However, their safety profile can be challenging to manage, thus limiting their clinical use [20]. For example, when azacitidine was taken for a phase 3 trial as a treatment of acute myeloid leukemia (AML), more than 40% of patients had Grade 3/4 neutropenia. In contrast, over 20% had Grade 3/4 thrombocytopenia [20]. Furthermore, nucleoside analogues have been shown to have low biological activity with high toxicity, affecting organs such as the heart and liver due to DNA hypomethylation [177]. Pharmaco-epigenetics and systems pharmacology are still far away from being applied in clinical settings, mainly because the relationship between DNA methylation, histone acetylation, and drug response and toxicity is poorly understood due to less research on this aspect compared to genomics [178].

Above all, since the epigenetic mechanisms are reversible, it can be said that this phenomenon allows the establishment of new approaches for preventing and treating obesity and T2DM; nevertheless, it is still a challenge to find ways of converting basic research into clinical application [44]. Thus, till now, no epigenetic drugs have received approval in treating metabolic diseases [24] since they have the potential to negatively alter the off-target genes [136]. Therefore, it becomes important to further conduct research on the role of epigenetic medications in metabolic diseases. Gaining enough knowledge about the epigenetic pathways underlying metabolic illnesses would be essential for developing innovative programs and concepts for their diagnosis, prevention, and treatment of metabolic diseases [24]. Additionally, it should also be noted that there are some challenges in identifying the source and effect of the relations between epigenetic markers, medical conditions, and drug responses since it is not straightforward sometimes [108]. Therefore, a lot still remains to be established about the directionality between epigenetics and drug response.

## 6. Conclusions and Future Perspectives

Epigenetics offers valuable insights into the pathogenesis of complex diseases and holds promise for identifying biomarkers for diagnosis, risk stratification, and therapeutic targeting. Despite its potential, current epigenetic therapies face challenges such as low specificity and associated toxicity, which may lead to adverse effects [20]. Future strategies could benefit from the development of more selective approaches, including epigenetic editing tools that enable precise reprogramming of gene expression by modifying the local epigenetic landscape without altering the underlying DNA sequence [179].

Given the reversible nature of epigenetic modifications, particularly DNA methylation, histone modifications, and non-coding RNA regulation, these marks present promising targets for the prediction, prevention, and treatment of complex diseases such as obesity and T2DM [33,145]. For example, aberrant methylation of CpG islands in promoter regions has been linked to gene silencing and resistance to chemotherapy and radiation [180], indicating that targeting these regions may be a key focus of epigenetic-based therapy.

While some epigenetic drugs have received FDA approval and show promise in treating diseases such as cancer [20], further research is needed to elucidate the mechanisms by which genetic predisposition and environmental exposures interact to influence DNA methylation and the development of T2DM [47]. Although pharmaco-epigenetics presents a potentially transformative avenue for personalized medicine, its clinical application remains limited, and more translational research is required before it can be fully implemented in managing metabolic diseases [181].

## Data Availability

This is a comprehensive review article. Data sharing is not applicable to this article as no datasets were generated or analysed during the current study.

## Abbreviations

ROS	Reactive Oxygen Species
DM	Diabetes Mellitus
FDA	Food and Drug Administration
T2DM	Type 2 Diabetes Mellitus
T1DM	Type 1 Diabetes Mellitus
HMTs	Histone Methyltransferases
HDMs	Histone Demethylases
TET	Ten-Eleven Translocation
SAM	S-Adenosyl-L-methionine
5meC	5-Methylcytosine
5hmC	5-Hydroxymethylcytosine
5fC	5-Formylcytosine
5caC	5-Carboxylcytosine
DNMT	DNA Methyltransferase
Ac	Acetylation
Me	Methylation
P	Phosphorylation
NAFLD	Non-Alcoholic Fatty Liver Disease
Ub	Ubiquitination
Ser	Serotonylation
Cr	Crotonylation
IR	Insulin Resistance
HFD	High-Fat Diet
VPA	Valproic Acid
STAC	Sirtuin-Activating Compounds
